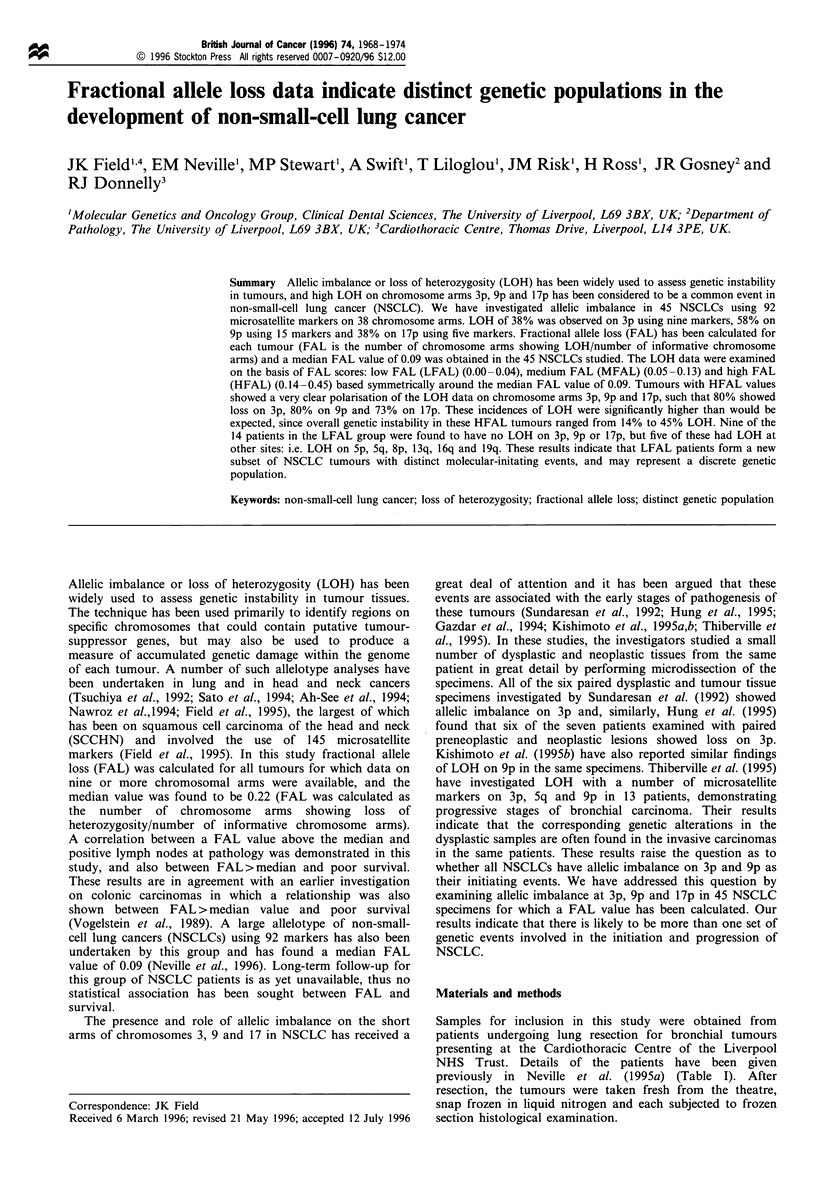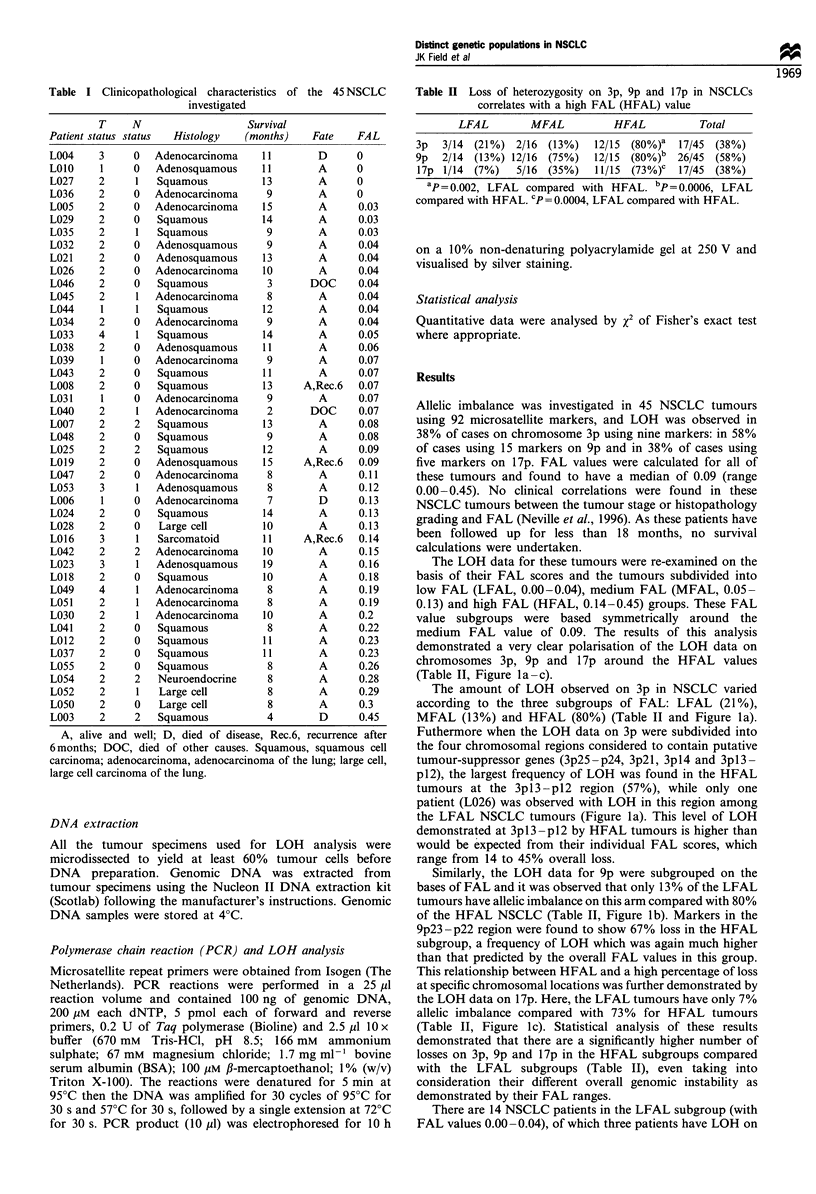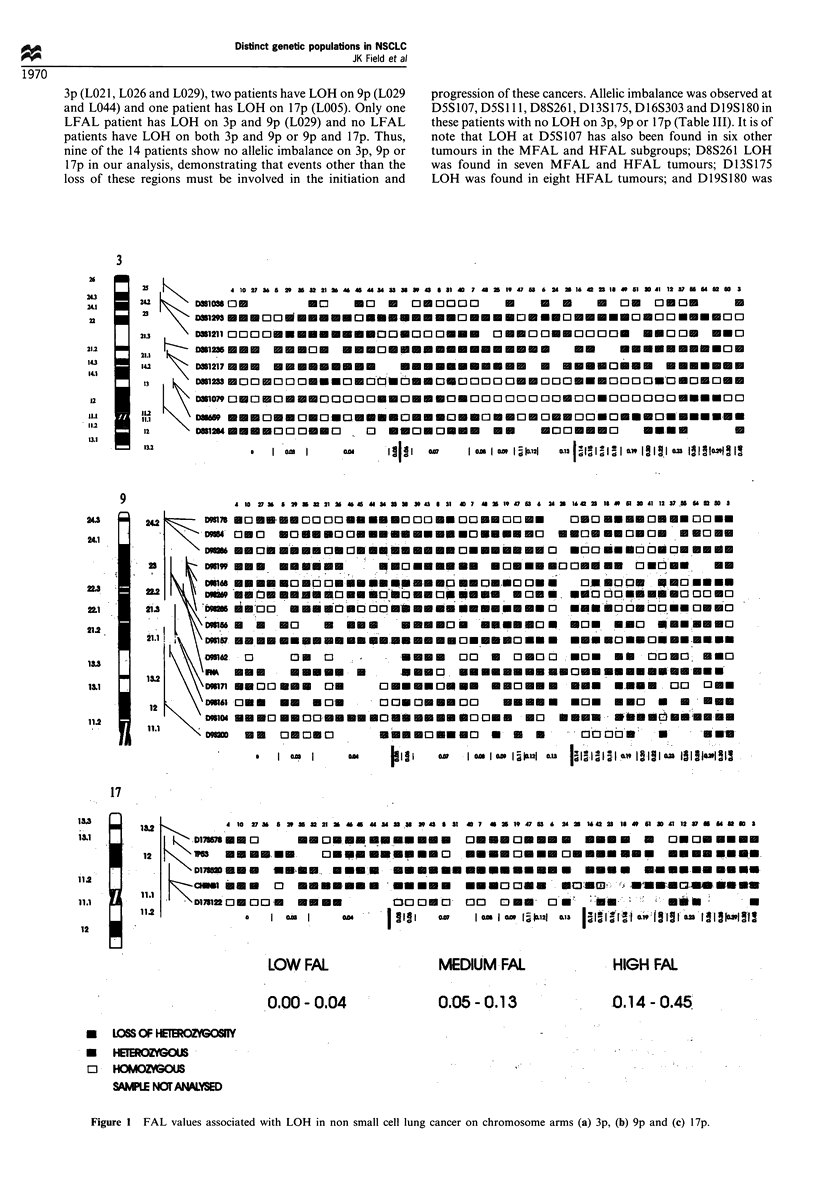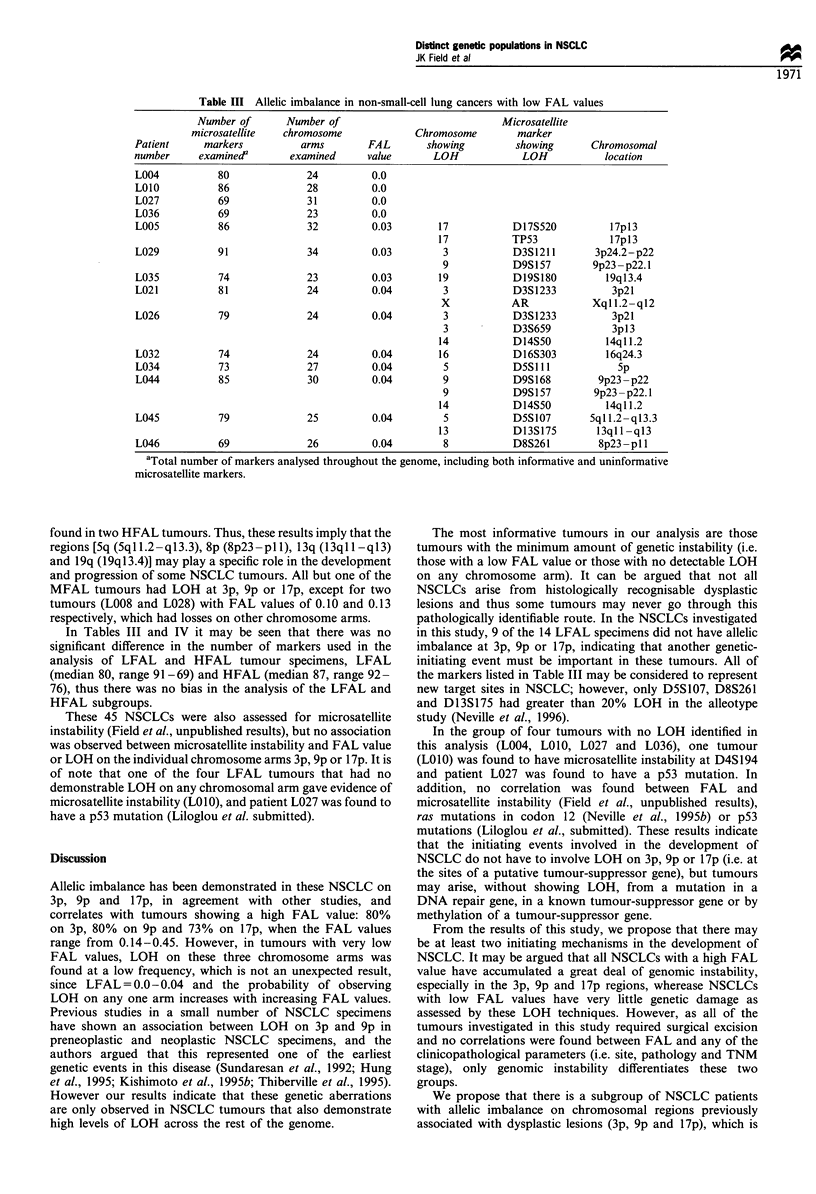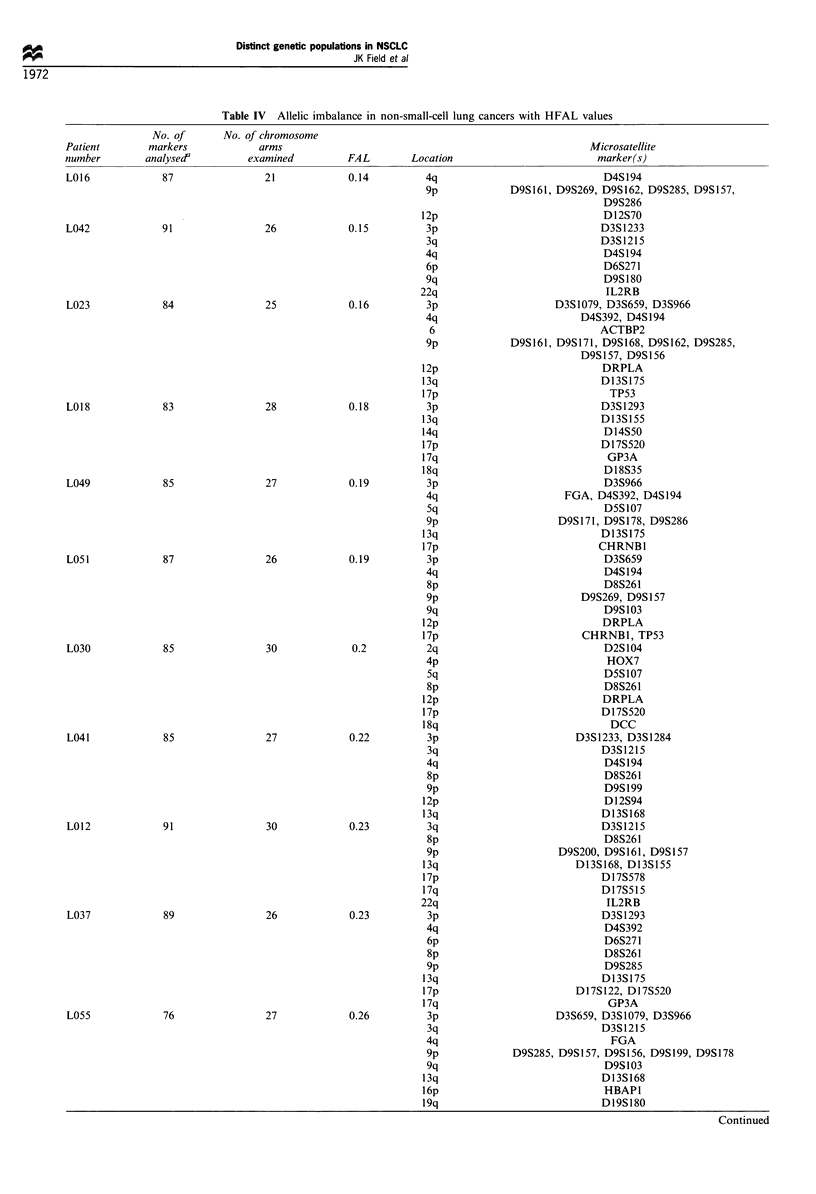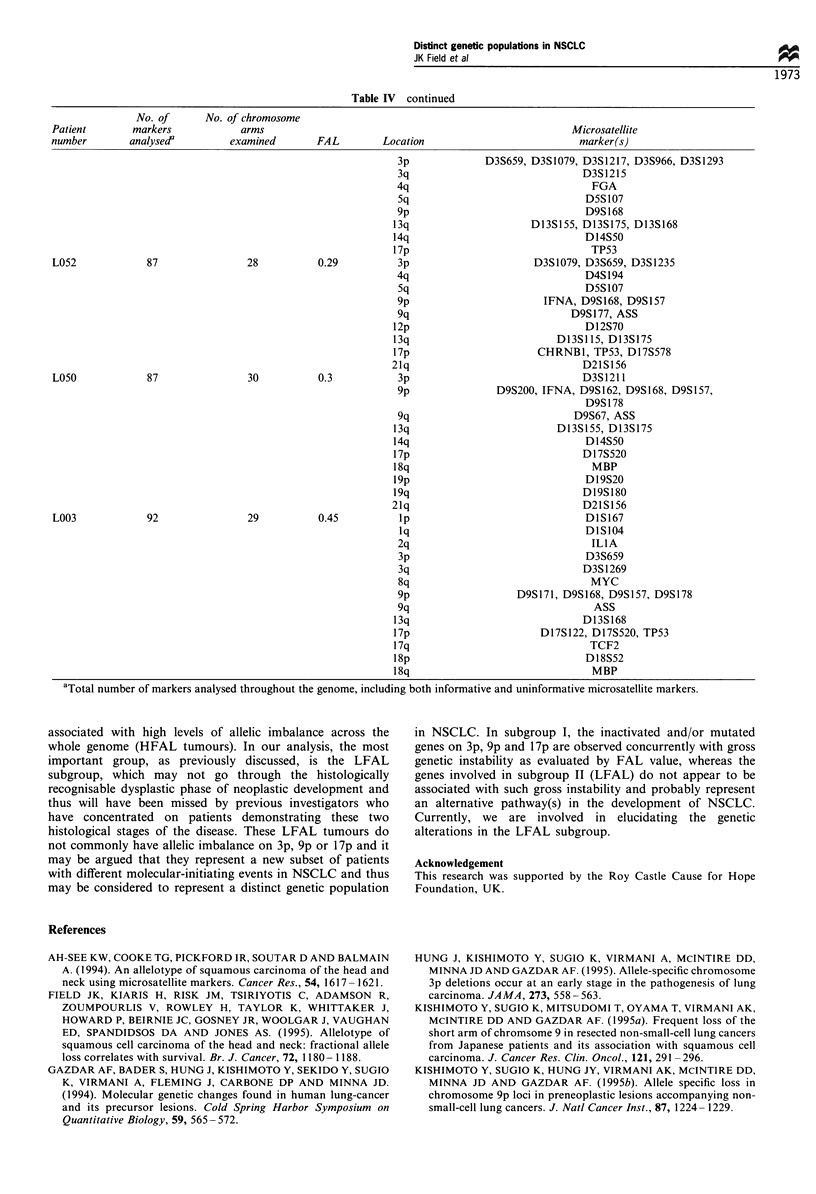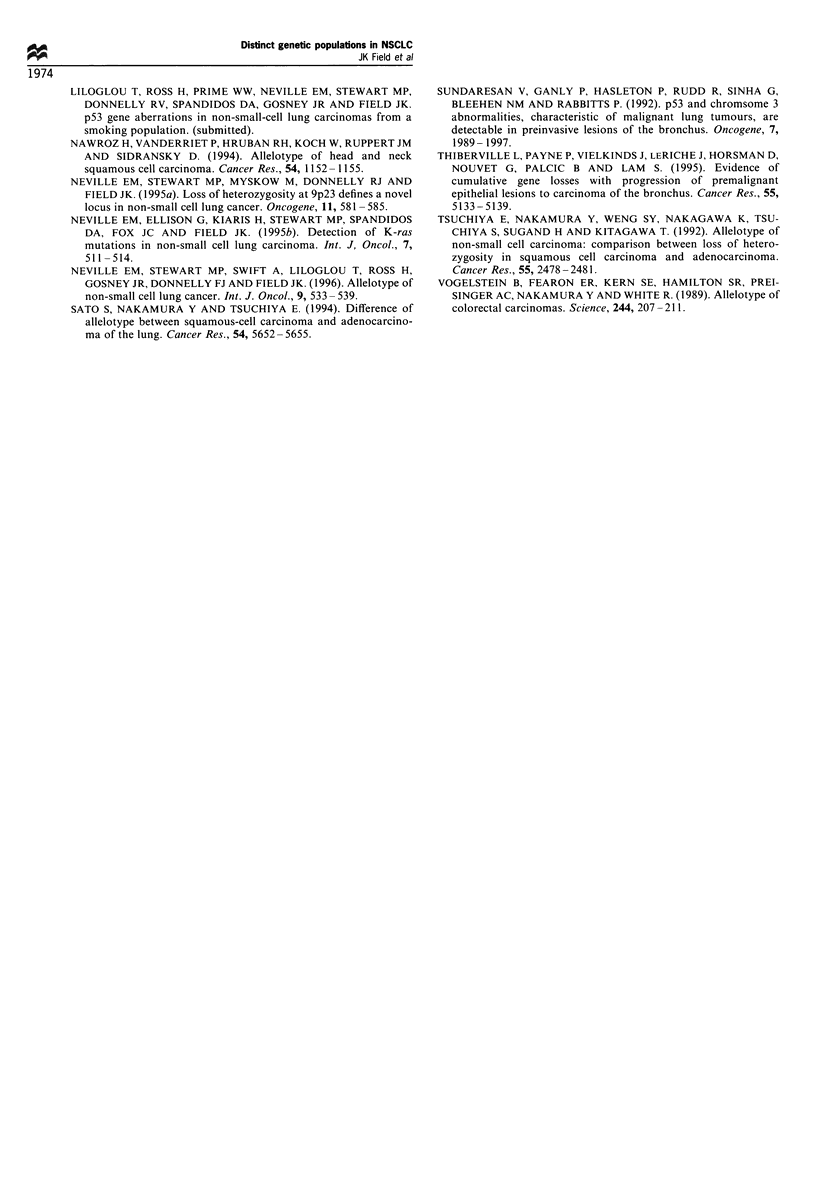# Fractional allele loss data indicate distinct genetic populations in the development of non-small-cell lung cancer.

**DOI:** 10.1038/bjc.1996.661

**Published:** 1996-12

**Authors:** J. K. Field, E. M. Neville, M. P. Stewart, A. Swift, T. Liloglou, J. M. Risk, H. Ross, J. R. Gosney, R. J. Donnelly

**Affiliations:** Molecular Genetics and Oncology Group, University of Liverpool, UK.

## Abstract

Allelic imbalance or loss of heterozygosity (LOH) has been widely used to assess genetic instability in tumours, and high LOH on chromosome arms 3p, 9p and 17p has been considered to be a common event in non-small-cell lung cancer (NSCLC). We have investigated allelic imbalance in 45 NSCLCs using 92 microsatellite markers on 38 chromosome arms. LOH of 38% was observed on 3p using nine markers, 58% on 9p using 15 markers and 38% on 17p using five markers. Fractional allele loss (FAL) has been calculated for each tumour (FAL is the number of chromosome arms showing LOH/number of informative chromosome arms) and a median FAL value of 0.09 was obtained in the 45 NSCLCs studied. The LOH data were examined on the basis of FAL scores: low FAL (LFAL) (0.00-0.04), medium FAL (MFAL) (0.05-0.13) and high FAL (HFAL) (0.14-0.45) based symmetrically around the median FAL value of 0.09. Tumours with HFAL values showed a very clear polarisation of the LOH data on chromosome arms 3p, 9p and 17p, such that 80% showed loss on 3p, 80% on 9p and 73% on 17p. These incidences of LOH were significantly higher than would be expected, since overall genetic instability in these HFAL tumours ranged from 14% to 45% LOH. Nine of the 14 patients in the LFAL group were found to have no LOH on 3p, 9p or 17p, but five of these had LOH at other sites: i.e. LOH on 5p, 5q, 8p, 13q, 16q and 19q. These results indicate that LFAL patients form a new subset of NSCLC tumours with distinct molecular-initating events, and may represent a discrete genetic population.


British Journal of Cancer (1996) 74, 1968-1974
© 1996 Stockton Press All rights reserved 0007-0920/96 $12.00

### Fractional allele loss data indicate distinct genetic populations in the development of non-small-cell lung cancer

JK Field[1,4], EM Neville[1], MP Stewart[1], A Swift[1], T Liloglou[1], JM Risk[1], H Ross[1], JR Gosney[2] and RJ Donnelly[3]

[1]*Molecular Genetics and Oncology Group, Clinical Dental Sciences, The University of Liverpool, L69 3BX, UK;* [2]*Department of Pathology, The University of Liverpool, L69 3BX, UK;* [3]*Cardiothoracic Centre, Thomas Drive, Liverpool, L14 3PE, UK.*

**Summary** Allelic imbalance or loss of heterozygosity (LOH) has been widely used to assess genetic instability in tumours, and high LOH on chromosome arms 3p, 9p and 17p has been considered to be a common event in non-small-cell lung cancer (NSCLC). We have investigated allelic imbalance in 45 NSCLCs using 92 microsatellite markers on 38 chromosome arms. LOH of 38% was observed on 3p using nine markers, 58% on 9p using 15 markers and 38% on 17p using five markers. Fractional allele loss (FAL) has been calculated for each tumour (FAL is the number of chromosome arms showing LOH/number of informative chromosome arms) and a median FAL value of 0.09 was obtained in the 45 NSCLCs studied. The LOH data were examined on the basis of FAL scores: low FAL (LFAL) (0.00–0.04), medium FAL (MFAL) (0.05–0.13) and high FAL (HFAL) (0.14–0.45) based symmetrically around the median FAL value of 0.09. Tumours with HFAL values showed a very clear polarisation of the LOH data on chromosome arms 3p, 9p and 17p, such that 80% showed loss on 3p, 80% on 9p and 73% on 17p. These incidences of LOH were significantly higher than would be expected, since overall genetic instability in these HFAL tumours ranged from 14% to 45% LOH. Nine of the 14 patients in the LFAL group were found to have no LOH on 3p, 9p or 17p, but five of these had LOH at other sites: i.e. LOH on 5p, 5q, 8p, 13q, 16q and 19q. These results indicate that LFAL patients form a new subset of NSCLC tumours with distinct molecular-initating events, and may represent a discrete genetic population.

**Keywords:** non-small-cell lung cancer; loss of heterozygosity; fractional allele loss; distinct genetic population

Allelic imbalance or loss of heterozygosity (LOH) has been widely used to assess genetic instability in tumour tissues. The technique has been used primarily to identify regions on specific chromosomes that could contain putative tumour-suppressor genes, but may also be used to produce a measure of accumulated genetic damage within the genome of each tumour. A number of such allelotype analyses have been undertaken in lung and in head and neck cancers (Tsuchiya *et al.*, 1992; Sato *et al.*, 1994; Ah-See *et al.*, 1994; Nawroz *et al.*,1994; Field *et al.*, 1995), the largest of which has been on squamous cell carcinoma of the head and neck (SCCHN) and involved the use of 145 microsatellite markers (Field *et al.*, 1995). In this study fractional allele loss (FAL) was calculated for all tumours for which data on nine or more chromosomal arms were available, and the median value was found to be 0.22 (FAL was calculated as the number of chromosome arms showing loss of heterozygosity/number of informative chromosome arms). A correlation between a FAL value above the median and positive lymph nodes at pathology was demonstrated in this study, and also between FAL>median and poor survival. These results are in agreement with an earlier investigation on colonic carcinomas in which a relationship was also shown between FAL>median value and poor survival (Vogelstein *et al.*, 1989). A large allelotype of non-small-cell lung cancers (NSCLCs) using 92 markers has also been undertaken by this group and has found a median FAL value of 0.09 (Neville *et al.*, 1996). Long-term follow-up for this group of NSCLC patients is as yet unavailable, thus no statistical association has been sought between FAL and survival.

The presence and role of allelic imbalance on the short arms of chromosomes 3, 9 and 17 in NSCLC has received a great deal of attention and it has been argued that these events are associated with the early stages of pathogenesis of these tumours (Sundaresan *et al.*, 1992; Hung *et al.*, 1995; Gazdar *et al.*, 1994; Kishimoto *et al.*, 1995*a*,*b*; Thiberville *et al.*, 1995). In these studies, the investigators studied a small number of dysplastic and neoplastic tissues from the same patient in great detail by performing microdissection of the specimens. All of the six paired dysplastic and tumour tissue specimens investigated by Sundaresan *et al.* (1992) showed allelic imbalance on 3p and, similarly, Hung *et al.* (1995) found that six of the seven patients examined with paired preneoplastic and neoplastic lesions showed loss on 3p. Kishimoto *et al.* (1995*b*) have also reported similar findings of LOH on 9p in the same specimens. Thiberville *et al.* (1995) have investigated LOH with a number of microsatellite markers on 3p, 5q and 9p in 13 patients, demonstrating progressive stages of bronchial carcinoma. Their results indicate that the corresponding genetic alterations in the dysplastic samples are often found in the invasive carcinomas in the same patients. These results raise the question as to whether all NSCLCs have allelic imbalance on 3p and 9p as their initiating events. We have addressed this question by examining allelic imbalance at 3p, 9p and 17p in 45 NSCLC specimens for which a FAL value has been calculated. Our results indicate that there is likely to be more than one set of genetic events involved in the initiation and progression of NSCLC.

### Materials and methods

Samples for inclusion in this study were obtained from patients undergoing lung resection for bronchial tumours presenting at the Cardiothoracic Centre of the Liverpool NHS Trust. Details of the patients have been given previously in Neville *et al.* (1995*a*) (Table I). After resection, the tumours were taken fresh from the theatre, snap frozen in liquid nitrogen and each subjected to frozen section histological examination.

Correspondence: JK Field
Received 6 March 1996; revised 21 May 1996; accepted 12 July 1996

**Table I** Clinicopathological characteristics of the 45 NSCLC investigated

| Patient | T status | N status | Histology | Survival (months) | Fate | FAL |
|---|---|---|---|---|---|---|
| L004 | 3 | 0 | Adenocarcinoma | 11 | D | 0 |
| L010 | 1 | 0 | Adenosquamous | 11 | A | 0 |
| L027 | 2 | 1 | Squamous | 13 | A | 0 |
| L036 | 2 | 0 | Adenocarcinoma | 9 | A | 0 |
| L005 | 2 | 0 | Adenocarcinoma | 15 | A | 0.03 |
| L029 | 2 | 0 | Squamous | 14 | A | 0.03 |
| L035 | 2 | 1 | Squamous | 9 | A | 0.03 |
| L032 | 2 | 0 | Adenosquamous | 9 | A | 0.04 |
| L021 | 2 | 0 | Adenosquamous | 13 | A | 0.04 |
| L026 | 2 | 0 | Adenocarcinoma | 10 | A | 0.04 |
| L046 | 2 | 0 | Squamous | 3 | DOC | 0.04 |
| L045 | 2 | 1 | Adenocarcinoma | 8 | A | 0.04 |
| L044 | 1 | 1 | Squamous | 12 | A | 0.04 |
| L034 | 2 | 0 | Adenocarcinoma | 9 | A | 0.04 |
| L033 | 4 | 1 | Squamous | 14 | A | 0.05 |
| L038 | 2 | 0 | Adenosquamous | 11 | A | 0.06 |
| L039 | 1 | 0 | Adenocarcinoma | 9 | A | 0.07 |
| L043 | 2 | 0 | Squamous | 11 | A | 0.07 |
| L008 | 2 | 0 | Squamous | 13 | A,Rec.6 | 0.07 |
| L031 | 1 | 0 | Adenocarcinoma | 9 | A | 0.07 |
| L040 | 2 | 1 | Adenocarcinoma | 2 | DOC | 0.07 |
| L007 | 2 | 2 | Squamous | 13 | A | 0.08 |
| L048 | 2 | 0 | Squamous | 9 | A | 0.08 |
| L025 | 2 | 2 | Squamous | 12 | A | 0.09 |
| L019 | 2 | 0 | Adenosquamous | 15 | A,Rec.6 | 0.09 |
| L047 | 2 | 0 | Adenocarcinoma | 8 | A | 0.11 |
| L053 | 3 | 1 | Adenosquamous | 8 | A | 0.12 |
| L006 | 1 | 0 | Adenocarcinoma | 7 | D | 0.13 |
| L024 | 2 | 0 | Squamous | 14 | A | 0.13 |
| L028 | 2 | 0 | Large cell | 10 | A | 0.13 |
| L016 | 3 | 1 | Sarcomatoid | 11 | A,Rec.6 | 0.14 |
| L042 | 2 | 2 | Adenocarcinoma | 10 | A | 0.15 |
| L023 | 3 | 1 | Adenosquamous | 19 | A | 0.16 |
| L018 | 2 | 0 | Squamous | 10 | A | 0.18 |
| L049 | 4 | 1 | Adenocarcinoma | 8 | A | 0.19 |
| L051 | 2 | 1 | Adenocarcinoma | 8 | A | 0.19 |
| L030 | 2 | 1 | Adenocarcinoma | 10 | A | 0.2 |
| L041 | 2 | 0 | Squamous | 8 | A | 0.22 |
| L012 | 2 | 0 | Squamous | 11 | A | 0.23 |
| L037 | 2 | 0 | Squamous | 11 | A | 0.23 |
| L055 | 2 | 0 | Squamous | 8 | A | 0.26 |
| L054 | 2 | 2 | Neuroendocrine | 8 | A | 0.28 |
| L052 | 2 | 1 | Large cell | 8 | A | 0.29 |
| L050 | 2 | 0 | Large cell | 8 | A | 0.3 |
| L003 | 2 | 2 | Squamous | 4 | D | 0.45 |

A, alive and well; D, died of disease, Rec.6, recurrence after 6 months; DOC, died of other causes. Squamous, squamous cell carcinoma; adenocarcinoma, adenocarcinoma of the lung; large cell, large cell carcinoma of the lung.

*DNA extraction*

All the tumour specimens used for LOH analysis were microdissected to yield at least 60% tumour cells before DNA preparation. Genomic DNA was extracted from tumour specimens using the Nucleon II DNA extraction kit (Scotlab) following the manufacturer's instructions. Genomic DNA samples were stored at 4°C.

*Polymerase chain reaction (PCR) and LOH analysis*

Microsatellite repeat primers were obtained from Isogen (The Netherlands). PCR reactions were performed in a 25 $\mu l$ reaction volume and contained 100 ng of genomic DNA, 200 $\mu M$ each dNTP, 5 pmol each of forward and reverse primers, 0.2 U of *Taq* polymerase (Bioline) and 2.5 $\mu l$ 10× buffer (670 mM Tris-HCl, pH 8.5; 166 mM ammonium sulphate; 67 mM magnesium chloride; 1.7 mg $ml^{-1}$ bovine serum albumin (BSA); 100 $\mu M$ $\beta$-mercaptoethanol; 1% (w/v) Triton X-100). The reactions were denatured for 5 min at 95°C then the DNA was amplified for 30 cycles of 95°C for 30 s and 57°C for 30 s, followed by a single extension at 72°C for 30 s. PCR product (10 $\mu l$) was electrophoresed for 10 h on a 10% non-denaturing polyacrylamide gel at 250 V and visualised by silver staining.

**Table II** Loss of heterozygosity on 3p, 9p and 17p in NSCLCs correlates with a high FAL (HFAL) value

| | LFAL | | MFAL | | HFAL | | Total | |
|---|---|---|---|---|---|---|---|---|
| 3p | 3/14 | (21%) | 2/16 | (13%) | 12/15 | (80%)[a] | 17/45 | (38%) |
| 9p | 2/14 | (13%) | 12/16 | (75%) | 12/15 | (80%)[b] | 26/45 | (58%) |
| 17p | 1/14 | (7%) | 5/16 | (35%) | 11/15 | (73%)[c] | 17/45 | (38%) |

[a]$P=0.002$, LFAL compared with HFAL. [b]$P=0.0006$, LFAL compared with HFAL. [c]$P=0.0004$, LFAL compared with HFAL.

*Statistical analysis*

Quantitative data were analysed by $\chi^2$ of Fisher's exact test where appropriate.

### Results

Allelic imbalance was investigated in 45 NSCLC tumours using 92 microsatellite markers, and LOH was observed in 38% of cases on chromosome 3p using nine markers: in 58% of cases using 15 markers on 9p and in 38% of cases using five markers on 17p. FAL values were calculated for all of these tumours and found to have a median of 0.09 (range 0.00–0.45). No clinical correlations were found in these NSCLC tumours between the tumour stage or histopathology grading and FAL (Neville *et al.*, 1996). As these patients have been followed up for less than 18 months, no survival calculations were undertaken.

The LOH data for these tumours were re-examined on the basis of their FAL scores and the tumours subdivided into low FAL (LFAL, 0.00–0.04), medium FAL (MFAL, 0.05–0.13) and high FAL (HFAL, 0.14–0.45) groups. These FAL value subgroups were based symmetrically around the medium FAL value of 0.09. The results of this analysis demonstrated a very clear polarisation of the LOH data on chromosomes 3p, 9p and 17p around the HFAL values (Table II, Figure 1a–c).

The amount of LOH observed on 3p in NSCLC varied according to the three subgroups of FAL: LFAL (21%), MFAL (13%) and HFAL (80%) (Table II and Figure 1a). Futhermore when the LOH data on 3p were subdivided into the four chromosomal regions considered to contain putative tumour-suppressor genes (3p25–p24, 3p21, 3p14 and 3p13–p12), the largest frequency of LOH was found in the HFAL tumours at the 3p13–p12 region (57%), while only one patient (L026) was observed with LOH in this region among the LFAL NSCLC tumours (Figure 1a). This level of LOH demonstrated at 3p13–p12 by HFAL tumours is higher than would be expected from their individual FAL scores, which range from 14 to 45% overall loss.

Similarly, the LOH data for 9p were subgrouped on the bases of FAL and it was observed that only 13% of the LFAL tumours have allelic imbalance on this arm compared with 80% of the HFAL NSCLC (Table II, Figure 1b). Markers in the 9p23–p22 region were found to show 67% loss in the HFAL subgroup, a frequency of LOH which was again much higher than that predicted by the overall FAL values in this group. This relationship between HFAL and a high percentage of loss at specific chromosomal locations was further demonstrated by the LOH data on 17p. Here, the LFAL tumours have only 7% allelic imbalance compared with 73% for HFAL tumours (Table II, Figure 1c). Statistical analysis of these results demonstrated that there are a significantly higher number of losses on 3p, 9p and 17p in the HFAL subgroups compared with the LFAL subgroups (Table II), even taking into consideration their different overall genomic instability as demonstrated by their FAL ranges.

There are 14 NSCLC patients in the LFAL subgroup (with FAL values 0.00–0.04), of which three patients have LOH on

3p (L021, L026 and L029), two patients have LOH on 9p (L029 and L044) and one patient has LOH on 17p (L005). Only one LFAL patient has LOH on 3p and 9p (L029) and no LFAL patients have LOH on both 3p and 9p or 9p and 17p. Thus, nine of the 14 patients show no allelic imbalance on 3p, 9p or 17p in our analysis, demonstrating that events other than the loss of these regions must be involved in the initiation and progression of these cancers. Allelic imbalance was observed at D5S107, D5S111, D8S261, D13S175, D16S303 and D19S180 in these patients with no LOH on 3p, 9p or 17p (Table III). It is of note that LOH at D5S107 has also been found in six other tumours in the MFAL and HFAL subgroups; D8S261 LOH was found in seven MFAL and HFAL tumours; D13S175 LOH was found in eight HFAL tumours; and D19S180 was

**Figure 1** FAL values associated with LOH in non small cell lung cancer on chromosome arms (**a**) 3p, (**b**) 9p and (**c**) 17p.

**Table III** Allelic imbalance in non-small-cell lung cancers with low FAL values

| *Patient number* | *Number of microsatellite markers examined*[a] | *Number of chromosome arms examined* | *FAL value* | *Chromosome showing LOH* | *Microsatellite marker showing LOH* | *Chromosomal location* |
|---|---|---|---|---|---|---|
| L004 | 80 | 24 | 0.0 | | | |
| L010 | 86 | 28 | 0.0 | | | |
| L027 | 69 | 31 | 0.0 | | | |
| L036 | 69 | 23 | 0.0 | | | |
| L005 | 86 | 32 | 0.03 | 17 | D17S520 | 17p13 |
| | | | | 17 | TP53 | 17p13 |
| L029 | 91 | 34 | 0.03 | 3 | D3S1211 | 3p24.2–p22 |
| | | | | 9 | D9S157 | 9p23–p22.1 |
| L035 | 74 | 23 | 0.03 | 19 | D19S180 | 19q13.4 |
| L021 | 81 | 24 | 0.04 | 3 | D3S1233 | 3p21 |
| | | | | X | AR | Xq11.2–q12 |
| L026 | 79 | 24 | 0.04 | 3 | D3S1233 | 3p21 |
| | | | | 3 | D3S659 | 3p13 |
| | | | | 14 | D14S50 | 14q11.2 |
| L032 | 74 | 24 | 0.04 | 16 | D16S303 | 16q24.3 |
| L034 | 73 | 27 | 0.04 | 5 | D5S111 | 5p |
| L044 | 85 | 30 | 0.04 | 9 | D9S168 | 9p23–p22 |
| | | | | 9 | D9S157 | 9p23–p22.1 |
| | | | | 14 | D14S50 | 14q11.2 |
| L045 | 79 | 25 | 0.04 | 5 | D5S107 | 5q11.2–q13.3 |
| | | | | 13 | D13S175 | 13q11–q13 |
| L046 | 69 | 26 | 0.04 | 8 | D8S261 | 8p23–p11 |

[a]Total number of markers analysed throughout the genome, including both informative and uninformative microsatellite markers.

found in two HFAL tumours. Thus, these results imply that the regions [5q (5q11.2–q13.3), 8p (8p23–p11), 13q (13q11–q13) and 19q (19q13.4)] may play a specific role in the development and progression of some NSCLC tumours. All but one of the MFAL tumours had LOH at 3p, 9p or 17p, except for two tumours (L008 and L028) with FAL values of 0.10 and 0.13 respectively, which had losses on other chromosome arms.

In Tables III and IV it may be seen that there was no significant difference in the number of markers used in the analysis of LFAL and HFAL tumour specimens, LFAL (median 80, range 91–69) and HFAL (median 87, range 92–76), thus there was no bias in the analysis of the LFAL and HFAL subgroups.

These 45 NSCLCs were also assessed for microsatellite instability (Field *et al*., unpublished results), but no association was observed between microsatellite instability and FAL value or LOH on the individual chromosome arms 3p, 9p or 17p. It is of note that one of the four LFAL tumours that had no demonstrable LOH on any chromosomal arm gave evidence of microsatellite instability (L010), and patient L027 was found to have a p53 mutation (Liloglou *et al.* submitted).

### Discussion

Allelic imbalance has been demonstrated in these NSCLC on 3p, 9p and 17p, in agreement with other studies, and correlates with tumours showing a high FAL value: 80% on 3p, 80% on 9p and 73% on 17p, when the FAL values range from 0.14–0.45. However, in tumours with very low FAL values, LOH on these three chromosome arms was found at a low frequency, which is not an unexpected result, since LFAL = 0.0–0.04 and the probability of observing LOH on any one arm increases with increasing FAL values. Previous studies in a small number of NSCLC specimens have shown an association between LOH on 3p and 9p in preneoplastic and neoplastic NSCLC specimens, and the authors argued that this represented one of the earliest genetic events in this disease (Sundaresan *et al*., 1992; Hung *et al*., 1995; Kishimoto *et al*., 1995*b*; Thiberville *et al*., 1995). However our results indicate that these genetic aberrations are only observed in NSCLC tumours that also demonstrate high levels of LOH across the rest of the genome.

The most informative tumours in our analysis are those tumours with the minimum amount of genetic instability (i.e. those with a low FAL value or those with no detectable LOH on any chromosome arm). It can be argued that not all NSCLCs arise from histologically recognisable dysplastic lesions and thus some tumours may never go through this pathologically identifiable route. In the NSCLCs investigated in this study, 9 of the 14 LFAL specimens did not have allelic imbalance at 3p, 9p or 17p, indicating that another genetic-initiating event must be important in these tumours. All of the markers listed in Table III may be considered to represent new target sites in NSCLC; however, only D5S107, D8S261 and D13S175 had greater than 20% LOH in the alleotype study (Neville *et al*., 1996).

In the group of four tumours with no LOH identified in this analysis (L004, L010, L027 and L036), one tumour (L010) was found to have microsatellite instability at D4S194 and patient L027 was found to have a p53 mutation. In addition, no correlation was found between FAL and microsatellite instability (Field *et al*., unpublished results), *ras* mutations in codon 12 (Neville *et al*., 1995*b*) or p53 mutations (Liloglou *et al*., submitted). These results indicate that the initiating events involved in the development of NSCLC do not have to involve LOH on 3p, 9p or 17p (i.e. at the sites of a putative tumour-suppressor gene), but tumours may arise, without showing LOH, from a mutation in a DNA repair gene, in a known tumour-suppressor gene or by methylation of a tumour-suppressor gene.

From the results of this study, we propose that there may be at least two initiating mechanisms in the development of NSCLC. It may be argued that all NSCLCs with a high FAL value have accumulated a great deal of genomic instability, especially in the 3p, 9p and 17p regions, wherease NSCLCs with low FAL values have very little genetic damage as assessed by these LOH techniques. However, as all of the tumours investigated in this study required surgical excision and no correlations were found between FAL and any of the clinicopathological parameters (i.e. site, pathology and TNM stage), only genomic instability differentiates these two groups.

We propose that there is a subgroup of NSCLC patients with allelic imbalance on chromosomal regions previously associated with dysplastic lesions (3p, 9p and 17p), which is

**Table IV** Allelic imbalance in non-small-cell lung cancers with HFAL values

| *Patient number* | *No. of markers analysed*[a] | *No. of chromosome arms examined* | *FAL* | *Location* | *Microsatellite marker(s)* |
|---|---|---|---|---|---|
| L016 | 87 | 21 | 0.14 | 4q | D4S194 |
| | | | | 9p | D9S161, D9S269, D9S162, D9S285, D9S157, D9S286 |
| | | | | 12p | D12S70 |
| L042 | 91 | 26 | 0.15 | 3p | D3S1233 |
| | | | | 3q | D3S1215 |
| | | | | 4q | D4S194 |
| | | | | 6p | D6S271 |
| | | | | 9q | D9S180 |
| | | | | 22q | IL2RB |
| L023 | 84 | 25 | 0.16 | 3p | D3S1079, D3S659, D3S966 |
| | | | | 4q | D4S392, D4S194 |
| | | | | 6 | ACTBP2 |
| | | | | 9p | D9S161, D9S171, D9S168, D9S162, D9S285, D9S157, D9S156 |
| | | | | 12p | DRPLA |
| | | | | 13q | D13S175 |
| | | | | 17p | TP53 |
| L018 | 83 | 28 | 0.18 | 3p | D3S1293 |
| | | | | 13q | D13S155 |
| | | | | 14q | D14S50 |
| | | | | 17p | D17S520 |
| | | | | 17q | GP3A |
| | | | | 18q | D18S35 |
| L049 | 85 | 27 | 0.19 | 3p | D3S966 |
| | | | | 4q | FGA, D4S392, D4S194 |
| | | | | 5q | D5S107 |
| | | | | 9p | D9S171, D9S178, D9S286 |
| | | | | 13q | D13S175 |
| | | | | 17p | CHRNB1 |
| L051 | 87 | 26 | 0.19 | 3p | D3S659 |
| | | | | 4q | D4S194 |
| | | | | 8p | D8S261 |
| | | | | 9p | D9S269, D9S157 |
| | | | | 9q | D9S103 |
| | | | | 12p | DRPLA |
| | | | | 17p | CHRNB1, TP53 |
| L030 | 85 | 30 | 0.2 | 2q | D2S104 |
| | | | | 4p | HOX7 |
| | | | | 5q | D5S107 |
| | | | | 8p | D8S261 |
| | | | | 12p | DRPLA |
| | | | | 17p | D17S520 |
| | | | | 18q | DCC |
| L041 | 85 | 27 | 0.22 | 3p | D3S1233, D3S1284 |
| | | | | 3q | D3S1215 |
| | | | | 4q | D4S194 |
| | | | | 8p | D8S261 |
| | | | | 9p | D9S199 |
| | | | | 12p | D12S94 |
| | | | | 13q | D13S168 |
| L012 | 91 | 30 | 0.23 | 3q | D3S1215 |
| | | | | 8p | D8S261 |
| | | | | 9p | D9S200, D9S161, D9S157 |
| | | | | 13q | D13S168, D13S155 |
| | | | | 17p | D17S578 |
| | | | | 17q | D17S515 |
| | | | | 22q | IL2RB |
| L037 | 89 | 26 | 0.23 | 3p | D3S1293 |
| | | | | 4q | D4S392 |
| | | | | 6p | D6S271 |
| | | | | 8p | D8S261 |
| | | | | 9p | D9S285 |
| | | | | 13q | D13S175 |
| | | | | 17p | D17S122, D17S520 |
| | | | | 17q | GP3A |
| L055 | 76 | 27 | 0.26 | 3p | D3S659, D3S1079, D3S966 |
| | | | | 3q | D3S1215 |
| | | | | 4q | FGA |
| | | | | 9p | D9S285, D9S157, D9S156, D9S199, D9S178 |
| | | | | 9q | D9S103 |
| | | | | 13q | D13S168 |
| | | | | 16p | HBAP1 |
| | | | | 19q | D19S180 |

Continued

Table IV continued

| Patient number | No. of markers analysed[a] | No. of chromosome arms examined | FAL | Location | Microsatellite marker(s) |
|---|---|---|---|---|---|
| | | | | 3p | D3S659, D3S1079, D3S1217, D3S966, D3S1293 |
| | | | | 3q | D3S1215 |
| | | | | 4q | FGA |
| | | | | 5q | D5S107 |
| | | | | 9p | D9S168 |
| | | | | 13q | D13S155, D13S175, D13S168 |
| | | | | 14q | D14S50 |
| | | | | 17p | TP53 |
| L052 | 87 | 28 | 0.29 | 3p | D3S1079, D3S659, D3S1235 |
| | | | | 4q | D4S194 |
| | | | | 5q | D5S107 |
| | | | | 9p | IFNA, D9S168, D9S157 |
| | | | | 9q | D9S177, ASS |
| | | | | 12p | D12S70 |
| | | | | 13q | D13S115, D13S175 |
| | | | | 17p | CHRNB1, TP53, D17S578 |
| | | | | 21q | D21S156 |
| L050 | 87 | 30 | 0.3 | 3p | D3S1211 |
| | | | | 9p | D9S200, IFNA, D9S162, D9S168, D9S157, D9S178 |
| | | | | 9q | D9S67, ASS |
| | | | | 13q | D13S155, D13S175 |
| | | | | 14q | D14S50 |
| | | | | 17p | D17S520 |
| | | | | 18q | MBP |
| | | | | 19p | D19S20 |
| | | | | 19q | D19S180 |
| | | | | 21q | D21S156 |
| L003 | 92 | 29 | 0.45 | 1p | D1S167 |
| | | | | 1q | D1S104 |
| | | | | 2q | IL1A |
| | | | | 3p | D3S659 |
| | | | | 3q | D3S1269 |
| | | | | 8q | MYC |
| | | | | 9p | D9S171, D9S168, D9S157, D9S178 |
| | | | | 9q | ASS |
| | | | | 13q | D13S168 |
| | | | | 17p | D17S122, D17S520, TP53 |
| | | | | 17q | TCF2 |
| | | | | 18p | D18S52 |
| | | | | 18q | MBP |

[a]Total number of markers analysed throughout the genome, including both informative and uninformative microsatellite markers.

associated with high levels of allelic imbalance across the whole genome (HFAL tumours). In our analysis, the most important group, as previously discussed, is the LFAL subgroup, which may not go through the histologically recognisable dysplastic phase of neoplastic development and thus will have been missed by previous investigators who have concentrated on patients demonstrating these two histological stages of the disease. These LFAL tumours do not commonly have allelic imbalance on 3p, 9p or 17p and it may be argued that they represent a new subset of patients with different molecular-initiating events in NSCLC and thus may be considered to represent a distinct genetic population in NSCLC. In subgroup I, the inactivated and/or mutated genes on 3p, 9p and 17p are observed concurrently with gross genetic instability as evaluated by FAL value, whereas the genes involved in subgroup II (LFAL) do not appear to be associated with such gross instability and probably represent an alternative pathway(s) in the development of NSCLC. Currently, we are involved in elucidating the genetic alterations in the LFAL subgroup.

**Acknowledgement**
This research was supported by the Roy Castle Cause for Hope Foundation, UK.

### References

AH-SEE KW, COOKE TG, PICKFORD IR, SOUTAR D AND BALMAIN A. (1994). An allelotype of squamous carcinoma of the head and neck using microsatellite markers. *Cancer Res.*, **54,** 1617–1621.

FIELD JK, KIARIS H, RISK JM, TSIRIYOTIS C, ADAMSON R, ZOUMPOURLIS V, ROWLEY H, TAYLOR K, WHITTAKER J, HOWARD P, BEIRNIE JC, GOSNEY JR, WOOLGAR J, VAUGHAN ED, SPANDIDSOS DA AND JONES AS. (1995). Allelotype of squamous cell carcinoma of the head and neck: fractional allele loss correlates with survival. *Br. J. Cancer*, **72,** 1180–1188.

GAZDAR AF, BADER S, HUNG J, KISHIMOTO Y, SEKIDO Y, SUGIO K, VIRMANI A, FLEMING J, CARBONE DP AND MINNA JD. (1994). Molecular genetic changes found in human lung-cancer and its precursor lesions. *Cold Spring Harbor Symposium on Quantitative Biology*, **59,** 565–572.

HUNG J, KISHIMOTO Y, SUGIO K, VIRMANI A, McINTIRE DD, MINNA JD AND GAZDAR AF. (1995). Allele-specific chromosome 3p deletions occur at an early stage in the pathogenesis of lung carcinoma. *JAMA*, **273,** 558–563.

KISHIMOTO Y, SUGIO K, MITSUDOMI T, OYAMA T, VIRMANI AK, McINTIRE DD AND GAZDAR AF. (1995*a*). Frequent loss of the short arm of chromsome 9 in resected non-small-cell lung cancers from Japanese patients and its association with squamous cell carcinoma. *J. Cancer Res. Clin. Oncol.*, **121,** 291–296.

KISHIMOTO Y, SUGIO K, HUNG JY, VIRMANI AK, McINTIRE DD, MINNA JD AND GAZDAR AF. (1995*b*). Allele specific loss in chromosome 9p loci in preneoplastic lesions accompanying non-small-cell lung cancers. *J. Natl Cancer Inst.*, **87,** 1224–1229.

LILOGLOU T, ROSS H, PRIME WW, NEVILLE EM, STEWART MP, DONNELLY RV, SPANDIDOS DA, GOSNEY JR AND FIELD JK. p53 gene aberrations in non-small-cell lung carcinomas from a smoking population. (submitted).

NAWROZ H, VANDERRIET P, HRUBAN RH, KOCH W, RUPPERT JM AND SIDRANSKY D. (1994). Allelotype of head and neck squamous cell carcinoma. *Cancer Res.*, **54,** 1152–1155.

NEVILLE EM, STEWART MP, MYSKOW M, DONNELLY RJ AND FIELD JK. (1995*a*). Loss of heterozygosity at 9p23 defines a novel locus in non-small cell lung cancer. *Oncogene*, **11,** 581–585.

NEVILLE EM, ELLISON G, KIARIS H, STEWART MP, SPANDIDOS DA, FOX JC AND FIELD JK. (1995*b*). Detection of K-*ras* mutations in non-small cell lung carcinoma. *Int. J, Oncol.*, **7,** 511–514.

NEVILLE EM, STEWART MP, SWIFT A, LILOGLOU T, ROSS H, GOSNEY JR, DONNELLY FJ AND FIELD JK. (1996). Allelotype of non-small cell lung cancer. *Int. J. Oncol.*, **9,** 533–539.

SATO S, NAKAMURA Y AND TSUCHIYA E. (1994). Difference of allelotype between squamous-cell carcinoma and adenocarcinoma of the lung. *Cancer Res.*, **54,** 5652–5655.

SUNDARESAN V, GANLY P, HASLETON P, RUDD R, SINHA G, BLEEHEN NM AND RABBITTS P. (1992). p53 and chromsome 3 abnormalities, characteristic of malignant lung tumours, are detectable in preinvasive lesions of the bronchus. *Oncogene*, **7,** 1989–1997.

THIBERVILLE L, PAYNE P, VIELKINDS J, LERICHE J, HORSMAN D, NOUVET G, PALCIC B AND LAM S. (1995). Evidence of cumulative gene losses with progression of premalignant epithelial lesions to carcinoma of the bronchus. *Cancer Res.*, **55,** 5133–5139.

TSUCHIYA E, NAKAMURA Y, WENG SY, NAKAGAWA K, TSUCHIYA S, SUGAND H AND KITAGAWA T. (1992). Allelotype of non-small cell carcinoma: comparison between loss of heterozygosity in squamous cell carcinoma and adenocarcinoma. *Cancer Res.*, **55,** 2478–2481.

VOGELSTEIN B, FEARON ER, KERN SE, HAMILTON SR, PREISINGER AC, NAKAMURA Y AND WHITE R. (1989). Allelotype of colorectal carcinomas. *Science*, **244,** 207–211.